# Adenovirus infection in children with acute lower respiratory tract infections in Beijing, China, 2007 to 2012

**DOI:** 10.1186/s12879-015-1126-2

**Published:** 2015-10-01

**Authors:** Chunyan Liu, Yan Xiao, Jing Zhang, Lili Ren, Jianguo Li, Zhengde Xie, Baoping Xu, Yan Yang, Suyun Qian, Jianwei Wang, Kunling Shen

**Affiliations:** Beijing Children’s Hospital, Capital Medical University, Beijing, 100045 P. R. China; Beijing Pediatric Research Institute, Beijing, 100045 P. R. China; MOH Key Laboratory of Systems Biology of Pathogens and Dr. Christophe Mérieux Laboratory, IPB, CAMS-Fondation Mérieux, Institute of Pathogen Biology (IPB), Chinese Academy of Medical Sciences (CAMS) & Peking Union Medical College, Beijing, 100730 P. R. China

**Keywords:** Adenovirus, Acute lower respiratory tract infection, Type, Children

## Abstract

**Background:**

Human adenoviruses (HAdV) play a significant role in pediatric respiratory tract infections. To date, over 60 types of HAdV have been identified. Here, HAdV types are characterized in children in the Beijing area with acute lower respiratory tract infections (ALRTIs) and the clinical features and laboratory findings of hospitalized HAdV-infected cases are described.

**Methods:**

Respiratory specimens were collected from pediatric patients with ALRTIs in the emergency department or from those admitted to Beijing Children’s Hospital between March 2007 and December 2012. Infections with common respiratory viruses were determined by PCR or RT-PCR. HAdV positive samples were further typed by PCR and sequencing.

**Results:**

Among 3356 patients with ALRTIs, 194 (5.8 %) were found to have HAdV infection. HAdV infection was primarily confined to children (88.35 %) less than 5 years of age. A total of 11 different types of HAdV were detected throughout the study period, with HAdV-B7 (49.0 %) and HAdV-B3 (26.3 %) as the most prevalent types, followed by HAdV-C2 (7.7 %) and HAdVC1 (4.6 %). Newly emerging and re-emergent types or variants, HAdV-B55 (*n* = 5), HAdV-C57 (*n* = 3), and HAdV-B14p1 (*n* = 1), were identified. Results also included the reported first case of co-infection with HAdV-C2 and HAdV-C57. Clinical entities of patients with single HAdV infection (*n* = 49) were similar to those with mixed HAdV/respiratory syncytial virus (RSV) infections (*n* = 41). Patients with HAdV-B7 infection had longer duration of fever and higher serum levels of muscle enzymes than HAdV-B3-infected patients.

**Conclusions:**

During the study period, HAdV-B7 and HAdV-B3 were the predominant types identified in pediatric ALRTIs. HAdV-B7 infection tends to have more severe clinical consequences. The presence of newly emerging types or variants and co-infection with different types of HAdV highlights the need for constant and close surveillance of HAdV infection.

## Background

Acute lower respiratory tract infections (ALRTIs) are the leading cause of pediatric morbidity and mortality worldwide, particularly in developing countries. In infants and young children, ALRTIs are most frequently caused by respiratory viruses. One such virus, human adenovirus (HAdV), plays a significant role in pediatric respiratory tract infections, accounting for 2–5 % of the overall respiratory illnesses and 4–10 % of the pneumonias [1, 2]. Although most cases are mild and indistinguishable from other viral causes, ALRTIs caused by HAdV can be severe, or even fatal, and are associated with the highest risk of long term respiratory sequelae [3]. Thus, HAdV-associated ALRTIs are of particular interest to both clinicians and researchers.

HAdV are responsible for a wide spectrum of clinical diseases, including respiratory illness (both upper and lower respiratory tract), pharyngoconjunctival fever, conjunctivitis, cystitis, gastroenteritis, and neurologic and venereal disease [4]. HAdV were first isolated in 1953 as respiratory pathogens [5, 6]. To date, over 60 types of HAdV have been identified and classified into seven species (A to G) [7–14]. Cases of severe infection, outbreaks in closed populations, and even epidemic outbreaks have been associated with the newly emerging or re-emergent types or variants [15–17].

Interestingly, different types of HAdV display various tissue tropisms that correlate with different clinical manifestations of infection. HAdV infections of the respiratory tract are predominantly caused by HAdV-B (including subspecies B1 and B2), HAdV-C, or HAdV-E. The predominant types vary among different countries and regions and they change over time because transmission of novel strains between countries or across continents may occur [18].

Type identification is critical to epidemiological surveillance, detection of new strains, and understanding of HAdV pathogenesis. However, because most clinical laboratories do not type the isolates, there is little published information about epidemiologic and clinical features of HAdV infections by type in children with ALRTIs. To identify HAdV types and species in children with ALRTIs in Beijing area and to characterize clinical features and laboratory findings of hospitalized HAdV-infected cases, respiratory specimens were collected from hospital-admitted pediatric patients with ALRTIs and typed HAdV positive samples using PCR and sequencing.

## Methods

### Ethics statement

The study protocol was approved by the Ethical Review Committee of Beijing Children’s Hospital. Individual written informed consent was obtained from the parents or guardians of all participants.

### Patients and clinical specimens

From March 2007 to December 2012, pediatric patients with ALRTIs who presented in emergency department or were admitted to respiratory department or intensive care unit, Beijing Children’s Hospital, were recruited for the study. The study site hospital is a tertiary comprehensive pediatric hospital with over 900 beds and more than twenty clinical departments. ALRTIs were defined as the presence of signs and symptoms of respiratory tract infection (i.e., fever, coughing, rhinorrhea, oropharyngeal hyperemia, swelling of tonsils), and lower respiratory signs (tachypnea, dyspnea, retractions, or wheezing/rales upon auscultation). The patients were diagnosed with bronchitis, bronchiolitis or pneumonia. Chest X-rays were taken for all patients and the criteria for diagnosing pneumonia are the presence of lung infiltrates indicated by chest radiography. Nasopharyngeal aspirate or throat swab specimens were collected in virus transport media from each patient. No repeated samples were collected from any patient. All samples were stored at −80 °C prior to use.

### Preparation of nucleic acids

Total nucleic acids (DNA and RNA) were extracted from 200 μl nasopharyngeal aspirate or throat swab specimens using the NucliSens easyMAG™ automated extraction system (bioMérieux, Marcy l’Etoile, France) according to the manufacturer’s instructions and eluted in 60 μl elution buffer.

### Detection of respiratory viruses

The presence of common respiratory viral agents, including parainfluenza virus (PIV) type 1–4, influenza virus (IFV), respiratory syncytial virus (RSV), human rhinovirus (HRV), enterovirus (EV), human coronavirus (HCoV 229E, NL63, HKU1, and OC43), human metapneumovirus (HMPV), human bocavirus (HBoV), and HAdV was determined by multiplex RT-PCR, single RT-PCR, or PCR assays as previously described [19, 20]. Blank virus transport media here served as a negative control for nucleic acid extraction and PCR.

### Molecular typing and phylogenetic analysis of HAdV

HAdV positive samples were further amplified using a nested PCR procedure that targeted hyper variable regions 1–6 of the hexon gene as described by Lu and Erdman [21]. Expected amplicons ranged from 688 bp to 821 bp (secondary amplification) in length. Sequencing was performed in both directions using the amplification primers.

Sequences were proof read and assembled using SeqMan software v7.1.0 (DNASTAR Inc., WI, U.S.). For assignment of molecular identity and identification of the closest match, sequence alignment was performed using the Basic Local Alignment Search Tool (BLAST) against NCBI GenBank database (http://www.ncbi.nlm.nih.gov).

### Clinical data collection

Clinical data were retrospectively recorded by careful analysis of patient medical files in Beijing Children’s Hospital, using a predefined Microsoft Excel spreadsheet. Patients’ demographic, clinical, and radiologic findings were collected.

### Statistical analyses

Continuous variables were summarized as means ± standard deviations (SD) or medians. For categorical variables, percentages of patients in each category were calculated. Differences between groups were assessed using Pearson’s Chi square test or Fisher’s exact test for categorical variables and the one way ANOVA, Independent-Samples *T* test, Mann–Whitney *U* test, and Kruskal-Wallis test for continuous variables. All analyses were performed using SPSS software, version 19.0 (IBM Corporation, NY, U.S.). All tests were calculated in a two-tailed manner and a *P* value of <0.05 was considered statistically significant.

## Results

### Frequency of HAdV in children with ALRTIs

From March 2007 through December 2012, a total of 3356 patients with ALRTIs (2766 with pneumonia, 309 with bronchitis and 281 with bronchiolitis) were enrolled in this study. The mean age of study participants was 3.87 ± 4.03 years (median 1 year; age range, 0.5 month to 17 years and 17 months). There were 2085 male participants with a male-to-female ratio of 1.64:1.

At least one respiratory virus was detected in nasopharyngeal aspirate or throat swab specimens of 2322 (69.2 %) enrolled participants. RSV (33.4 %) was the most commonly detected viral pathogen, followed by HRV (26.6 %) and PIV (13.6 %). One hundred and ninety-four patients (5.8 %, 194/3356) were found to have HAdV infection, representing 8.7 % (194/2232) of patients with positive respiratory samples. Male paticipants were more likely to be infected with HAdV (135 boys and 59 girls, male to female ratio = 2.3:1). The mean age of infection was 2.13 ± 2.68 years (median, 1 year; age range, 1 month to 15 years). Most of HAdV-infected cases (88.35 %) were under 5 years of age and the highest percentage of HAdV infections (42.47 %) occurred in infants (age group 0– < 1 year), followed by the age group 1– < 2 years (27.84 %).

Additionally, one or more other respiratory viruses were detected in 69.6 % (*n* = 135) of 194 HAdV-infected participants. Dual viral infection was identified in 75 cases, triple infection in 45 cases, quadruple in 13 and quintuple in 2. RSV (*n* = 56) was the most frequently co-detected virus, followed by HRV (*n* = 53) and PIV (*n* = 42). HBoV (*n* = 24), HCoV (*n* = 15), IFV (*n* = 8), EV (*n* = 6), and HMPV (*n* = 5) were also found to be co-infected with HAdV.

### Typing of HAdV

One hundred and ninety-four HAdV-positive specimens were all successfully typed by hexon gene amplifying and sequencing. Throughout the study period, four species (A, B, C, E) of HAdV, including 11 different types were identified. Additionally, HAdV-B7 (*n* = 95; 49.0 %) and HAdV-B3 (*n* = 51; 26.3 %), which belong to species B, were the most prevalent HAdV types, accounting for 75.3 % of all HAdV-associated infections. HAdV-C2 (7.7 %), HAdV-C1 (4.6 %), HAdV-C5 (3.6 %), HAdV-B55 (2.6 %), HAdV-C6 (1.5 %), HAdV-C57 (1.5 %), HAdV-A31 (1.0 %), HAdV-B14 (0.5 %), and HAdV-E4 (0.5 %) were also detected.

Interestingly, sequencing results from one specimen showed superimposed peaks in the chromatograms. To confirm the possibility of multiple HAdV strains in that sample, PCR products were cloned and sequenced further. Distinct hexon genes of different types (HAdV-C2 and HAdV-C57) were verified.

### Temporal distribution of HAdV

**Fig. 1:**
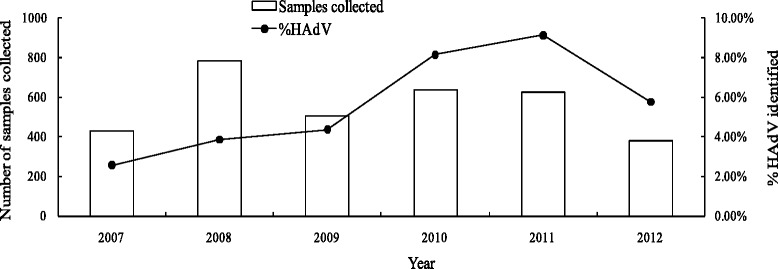
Samples collected and proportion of samples with HAdV identified in each year, 2007–2012

**Fig. 2:**
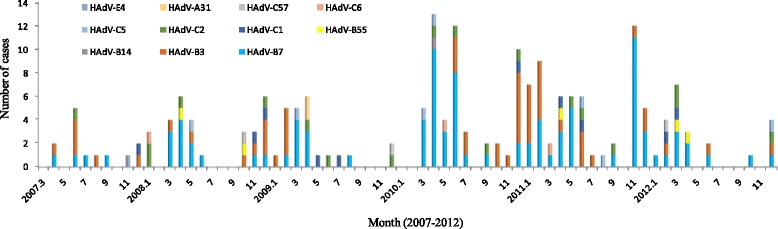
Seasonal distribution of HAdV infection in children with ALRTIs from 2007 to 2012. Detection numbers of different types of HAdV are shown in each month

HAdV detection rate varied through the years, ranging from 2.55 % in 2007 to 9.15 % in 2010 (Fig. 1). Additionally, Although HAdV was detected throughout the year, cases commonly peaked in winter and spring season (Fig. 2). Furthermore, different types of HAdV did not remain constant across the whole study period (Fig. 2). Specifically, HAdV-C1, −C2, −B3, and -B7 were detected throughout the study; HAdV-C5 in all years except 2007; HAdV-C6 and HAdV-C57 in years 2008, 2009, and 2012; HAdV-B55 in 2008, 2011, and 2012; and HAdV-E4, HAdV-A31, and HAdV-B14 in years 2007, 2009, and 2010, respectively.

### Clinical features of HAdV infections

Among the 194 HAdV-positive cases, 150 hospitalized cases were included in the clinical analysis, and 44 cases from the emergency department for which the details of the medical records were not available were excluded.

Pneumonia (*n* = 142, 94.7 %) was the most common clinical diagnosis, followed by bronchitis (*n* = 7) and bronchiolitis (*n* = 1). Additionally, almost all hospitalized HAdV-infected patients presented with fever (144/150, 96.0%) and coughing (149/150, 99.3 %) (Table 1). The mean peak body temperature was 39.53 ± 0.67 °C (*n* = 144, range 37.3 − 41.4 °C) and febrile seizures were noted in two febrile patients. In addition to respiratory symptoms, diarrhea, vomiting, skin rash, and conjunctivitis were noted in 21.3 %, 9.3 %, 10.0 % and 4.7 % of the patients respectively. Twenty-two patients (14.7 %) had underlying diseases, which included congenital heart disease (8 patients), airway anomaly (malacia, stenosis, 11 patients), bronchopulmonary dysplasia (1 patient), asthma (2 patients), or primary immunodeficiency (1 patient). Seventeen patients (11.3 %) required admission to the intensive care unit and 38 patients (11.3 %) received mechanical ventilation including both noninvasive (*n* = 31) and invasive (*n* = 7) modes. Analysis revealed that the mean value of white blood cell (WBC) count was 10.09 ± 4.61 × 10^9^/L (Table 2). Leukocytosis (WBC > 12.0 × 10^9^/L) was observed in 37 (24.7 %) patients. Eighty-one patients (54.0 %) had elevated serum C-reaction protein (CRP).

**Table 1:** Clinical manifestations and laboratory findings of HAdV infections in 150 hospitalized children with ALRTIs

		Group		
Variables	All cases	HAdV	HAdV/RSV	HAdV/RSV
		Single infection	Dual infections	Multiple infections
	(*n* = 150)	(*n* = 49)	(*n* = 18)	(*n* = 23)
Age (years)^a^	2.37 ± 2.89	3.68 ± 3.64	1.53 ± 2.41	1.20 ± 0.81
Male (%)	105 (70 %)	32 (65.3 %)	12 (66.7 %)	17 (73.9 %)
Symptoms and signs				
Fever	144 (96.0 %)	48 (98.0 %)	17 (94.4 %)	23 (100 %)
T max (°C)	39.53 ± 0.68	39.73 ± 0.66	39.48 ± 0.53	39.62 ± 0.73
Duration of fever (days)	15.31 ± 13.35	18.06 ± 18.85	14.18 ± 5.86	15.17 ± 7.25
Cough	149 (99.3 %)	49 (100 %)	18 (100 %)	23 (100 %)
Rhinorrhea	24 (16.0 %)	8 (16.3 %)	2 (11.1 %)	4 (17.4 %)
Wheezing	90 (60.0 %)	25 (51.0 %)	14 (77.8 %)	17 (73.9 %)
Swelling of tonsils	44 (29.3 %)	18 (36.7 %)	2 (11.1 %)	3 (13.0 %)
Rash	15 (10.0 %)	5 (10.2 %)	7 (12.3 %)	3 (6.8 %)
Vomitting	14 (9.3 %)	5 (10.2 %)	1 (5.6 %)	4 (17.4 %)
Diarrhea	32 (21.3 %)	11 (22.4 %)	3 (16.7 %)	7 (30.4 %)
Conjunctivitis	7 (4.7 %)	2 (4.1 %)	1 (5.6 %)	0 (0 %)
Dyspnea	61 (40.7 %)	18 (36.7 %)	8 (44.4 %)	12 (52.2 %)
ICU admission	17 (11.3 %)	7 (14.3 %)	2 (11.1 %)	5 (21.7 %)
Any underlying diseases	22 (14.7 %)	10 (20.4 %)	2 (11.1 %)	2 (8.7 %)
Treatment				
Mechanical ventilation	38 (25.3 %)	13 (26.5 %)	4 (22.2 %)	8 (34.8 %)
Coticosteriods	122 (81.3 %)	40 (81.6 %)	13 (72.2 %)	22 (95.7 %)
Inhaled Intravenous	74 (49.3 %)	22 (44.9 %)	8 (44.4 %)	14 (60.9 %)
Immunoglobulin	34 (22.7 %)	8 (16.3 %)	1 (5.6 %)	6 (26.1 %)
Length of hospital stay (days)	18.32 ± 11.31	16.19 ± 11.01	20.17 ± 10.22	20.52 ± 10.44
Laboratory findings				
WBC (×10^9^/L)	10.09 ± 4.61	9.34 ± 4.85	10.50 ± 3.79	9.86 ± 4.26
CRP (mg/L)	31.64 ± 35.93	36.01 ± 39.67	36.78 ± 30.25	32.42 ± 43.52
AST, median (IQR) (U/L)	33 (45–71.5)	45 (34–81.8)	38.5 (33–56.3)	57 (33–83)
ALT, median (IQR) (U/L)	21 (16–36)	18.1 (15–33)	21 (18–36.5)	22 (19–46)
LDH, median (IQR) (U/L)	360 (262.5-668.5)	369.5 (285.7-899.5)	326.5 (260–584.5)	439 (288–747)
CK, median (IQR) (U/L)	83 (45–192)	87.5 (46.8-264)	84 (42.5-221.8)	88 (38–137)
HBDH, median (IQR) (U/L)	274 (200.5-508.5)	275 (203.3-562.3)	258 (203–447.9)	355 (221.5–524)

*ICU* intensive care unit, *WBC* white blood cell, *CRP* C-reaction protein, *AST* aspartate aminotransferase, *ALT* alanine aminotransferase, LDH, lactate dehydrogenase, *CK* creatinine kinase, *HBDH* hydroxybutyrate dehydrogenase, *IQR* interquartile range

^a^one way ANOVA: single infection vs dual infections vs multiple infections, *p* = 0.001

**Table 2:** Clinical information for HAdV-B7 and HAdV-B3 infected children

	HAdV-B7		HAdV-B3	
Variables	Single infection	Coinfection	Single infection	Coinfection
	(*n* = 30)	(*n* = 52)	(*n* = 15)	(*n* = 27)
Age (years)^a^	3.47 ± 3.08	1.20 ± 1.26	4.91 ± 4.63	2.62 ± 2.99
Male (%)	19 (63.3 %)	41 (56.2 %)	9 (60.0 %)	17 (63.0 %)
Symptoms and signs				
Fever	29 (96.7 %)	51 (98.1 %)	15 (100 %)	24 (88.9 %)
T max (°C)	39.84 ± 0.71	39.50 ± 0.57	39.55 ± 0.53	39.78 ± 0.67
Duration of fever (days)^b^	22.07 ± 21.52	14.02 ± 6.36	9.73 ± 7.31	15.38 ± 14.36
Cough	30 (100 %)	52 (100 %)	15 (100 %)	26 (96.3 %)
Rhinorrhea	4 (13.3 %)	7 (13.5 %)	3 (20 %)	5 (18.5 %)
Wheezing	17 (56.7 %)	37 (71.2 %)	7 (46.7 %)	13 (48.1 %)
Swelling of tonsils	11 (36.7 %)	9 (17.3 %)	7 (46.7 %)	9 (33.3 %)
Rash	3 (10.0 %)	5 (9.6 %)	0	3 (11.1 %)
Vomitting	3 (10.0 %)	5 (9.6 %)	2 (13.3 %)	3 (11.1 %)
Diarrhea	9 (30.0 %)	13 (25.0 %)	1 (6.7 %)	7 (25.9 %)
Conjunctivitis	1 (3.3 %)	3 (5.8 %)	0	1 (3.7 %)
Dyspnea	13 (43.3 %)	26 (50.0 %)	4 (26.7 %)	9 (33.3 %)
ICU admission	6 (20.0 %)	7 (13.5 %)	0	2 (7.4 %)
Any underlying diseases	5 (16.7 %)	6 (11.5 %)	4 (26.7 %)	4 (14.8 %)
Treatment				
Mechanical ventilation	8 (26.7 %)	19 (36.5%)	4 (26.7 %)	4 (14.8 %)
Coticosteriods	26 (86.7 %)	45 (86.5%)	12 (80.0 %)	24 (88.9 %)
Inhaled	16 (53.3 %)	29 (55.8%)	5 (33.3 %)	14 (51.9 %)
Intravenous				
Immunoglobulin^c^	8 (26.7 %)	20 (38.5 %)	0	4 (14.8 %)
Length of hospital stay (days)	18.50 ± 12.87	22.44 ± 11.67	12.33 ± 4.95	16.89 ± 10.88
Laboratory findings				
WBC (×10^9^/L)	8.75 ± 4.63	9.96 ± 4.95	10.34 ± 5.23	10.87 ± 3.91
CRP (mg/L)	34.68 ± 42.70	33.95 ± 39.30	41.96 ± 36.84	31.54 ± 27.96
AST, median (IQR) (U/L)^d^	67 (38.8–100)	60 (38.3–94.3)	33 (27–45)	38 (26–51)
ALT, median (IQR) (U/L)^e^	20 (16–36)	25 (19–42.8)	15 (12–21)	17 (13–26)
LDH, median (IQR) (U/L)^f^	565 (341.8–1232.8)	470.5 (305.3–922.8)	297 (207–397)	311 (245–462)
CK, median (IQR) (U/L)	125.5 (48–499.8)	87 (45.5–187.5)	77 (46–99)	6 (43–115)
HBDH, median (IQR) (U/L)^g^	408 (242.8–803)	356.5 (225.9–647.5)	219 (162–313)	210 (199–374)

*ICU* intensive care unit, *WBC* white blood cell, *CRP* C-reaction protein, *AST* aspartate aminotransferase, *ALT* alanine aminotransferase, *LDH* lactate dehydrogenase, *CK* creatinine kinase, *HBDH* hydroxybutyrate dehydrogenase, *IQR* interquartile range

^a^Independent-Samples *T* test: HAdV-B7 single infection vs HAdV-B7 coinfection, *p* = 0.001

^b^Independent-Samples *T* test: HAdV-B7 single infection vs HAdV-B3 single infection, *p* = 0.038

^c^Fisher exact test: HAdV-B7 single infection vs HAdV-B3 single infecion, *p* = 0.038

^d^Mann–Whitney *U* test: HAdV-B7 single infection vs HAdV-B3 single infection, *p* < 0.001

^e^Mann–Whitney *U* test: HAdV-B7 single infection vs HAdV-B3 single infection, *p* = 0.029

^f^Mann–Whitney *U* test: HAdV-B7 single infection vs HAdV-B3 single infection, *p* = 0.001

^g^Mann–Whitney *U* test: HAdV-B7 single infection vs HAdV-B3 single infection, *p* = 0.001

Given RSV was the virus most frequently co-detected with HAdV, differences among patients with single HAdV infection (*n* = 49) and those with HAdV/RSV co-infections, including both dual infections (*n* = 18) and multiple infections (HAdV/RSV with one or more other respiratory viruses, *n* = 23), were assessed (Table 1). The mean age of patients with multiple infections (1.20 ± 0.81) and dual infections (1.53 ± 2.41) was significantly younger than those with single HAdV infection (3.68 ± 3.64) (*P* = 0.001). However clinical characteristics and laboratory findings showed no significant differences among different groups.

Because HAdV-B7 and HAdV-B3 were the most predominant type among patients with HAdV infection, the clinical entities of patients with single HAdV-B7 infection (*n* = 30) and those with single HAdV-B3 infection (*n* = 15) were also compared to exclude the possible effect of other respiratory virus infection (Table 2). Patients with single HAdV-B7 infection showed longer duration of fever (22.07 ± 21.52 vs 9.73 ± 7.31, *P* = 0.038) than patients with HAdV-B3 alone. Immunoglobulin was more frequently used in single HAdV-B7 infected patients than in single HAdV-B3 group (*P* = 0.038). Patients with HAdV-B7 alone also tend to require longer hospital stay (18.50 ± 12.87 vs 12.33 ± 4.95, *P* = 0.082) than those with single HAdV-B3 infection, although no significant difference was found. Biochemical tests demonstrated aspartate aminotransferase (AST), alanine aminotransferase (ALT), lactate dehydrogenase (LDH) and hydroxybutyrate dehydrogenase (HBDH) levels were significantly higher in the single HAdV-B7 infected group.

Two patients died in-hospital. Both of them required ICU admission and died of multiple organ failure. One was a 17 month-old boy with multiple underlying conditions of complex congenital heart disease and tracheobronchial malformation. The other was a previously healthy 18 month-old boy. Analysis indicated that both fatal patients were infected with HAdV-B7 but no other respiratory viruses.

## Discussion

HAdV is a significant causative agent of respiratory tract illnesses in both children and adults. Here, the molecular epidemiology and clinical features of HAdV associated pediatric ALRTIs in Beijing Children’s Hospital from March 2007 to December 2012 were described. Results showed that the HAdV infection rate in the current study population was 5.8 %, which was consistent with previous reports from China and other countries [22–24]. Results showed that most patients with HAdV infection were younger than 5 years (88.35 %), which is similar to numbers reported in previous studies [1, 22, 23, 25–27]. This may because the immune systems of young children are not well developed, which leaves them prone to more severe HAdV disease. This may also suggest that school-age children are exposed to the most common endemic types of HAdV early in life, thereby establishing a protective immunity resulting only in mild clinical symptoms, such that upper respiratory tract infection does not require care in an emergency department or hospital in this age group.

Over a period of 6 years, 11 different types of HAdV belonging to 4 species (HAdV-A, B, C, E) were identified in respiratory specimens from children with ALRTIs. HAdV-7 and HAdV-3 of species B comprised the most prevalent types and presented throughout the duration of the study. Although these results were consistent with previous reports from Korea and Argentina [22, 28, 29], investigations from Croatia, Peru, Canada, France showed that species C predominated [1, 25, 26, 30]. This difference in type prevalence may be attributed to difference in regions, year of study, and population recruited.

Notably, some newly emerging or re-emergent types or variants were here identified, although only in rare cases. Five patients were found to have HAdV-B55 (formerly named HAdV-11a), which is an uncommon re-emergent type that once caused an outbreak of respiratory tract infection in a senior high school in Shanxi Province, China in 2006, including one fatal case [15]. Subsequently, HAdV-B55 has been associated with several outbreaks of respiratory disease in other provinces in China [31]. An emerging variant, HAdV-B14p1 (formerly known as 14a), was also found. Recently, HAdV-B14p1 has been associated with several large outbreaks of acute respiratory infection, which included severe and even fatal cases in the United States and Europe [16, 32]. Additionally, in 2011, an outbreak of febrile respiratory illness that affected 43 students in Gansu Province, China was reported to be caused by HAdV-B14p1 [33]. One HAdV-B14 infected patient who presented with bronchopneumonia and required hospitalization in April 2010 was identified. By further sequencing the fiber gene (data not shown), this strain was confirmed to be HAdV-B14p1 because it contained a unique characteristic 6-nuleotid deletion in fiber knob region as reported by Kajon et al. [32]. Last, this is the first report of detection of HAdV-C57 in respiratory samples collected from pediatric patients with ALRTIs and the first of co-detection of HAdV-C57 with HAdVC-2. HAdV-C57 (formerly designated strain 16700) was first isolated from the feces of a healthy child as part of an acute flaccid paralysis surveillance program. Computational genomic and bioinformatic analysis showed HAdV-C57 to be a recombinant virus with fiber gene nearly identical to HAdV-C6 and a unique hexon distinct from all viruses in species HAdV-C [34, 35]. Out of the three HAdV-C57-infected cases identified here, one was a previously healthy 9-month-old male who presented with bronchopneumonia and conjunctivitis requiring hospitalization. Because only a small number of HAdV-C57 positive cases were found here and all were co-infected with other respiratory viruses, the pathogenic role of HAdV-C57 in respiratory infections will require further investigation.

HAdV type is traditionally determined by virus isolation and subsequently serum neutralization tests, in which antibodies raised against specific type are used to suppress cytopathic effects in tissue culture assays. By nature of its design, this test can only reveal the dominant type. By applying PCR-based identification targeting hexon or fiber genes, co-infections with multiple HAdV types (types from same or different species) have been reported in both immunocompromised and immunocompetent patients [28, 29, 36–38]. In current study, results showed that one specimen contained both HAdV-C2 and HAdV-C57 by cloned sequencing the PCR products. These were amplified directly from respiratory samples using universal primers of hexon gene. This co-infected phenomenon was confirmed using the fiber gene sequencing results with type-specific primers (data not shown). The specimen was collected from a previously healthy 2.7-year old boy, presenting with fever, coughing and seizure at emergency department on December 10, 2009. Co-infection of different HAdV types has never been reported in any previous studies of Mainland China. The clinical implications of such co-infection remain unclear, and its role in HAdV pathogenesis and evolution will require further study.

Consistent with the report from Guangzhou, Southern China [24], results here showed that 69.6% of HAdV-infected participants were co-infected with one or more other respiratory tract viruses and that RSV was the most frequently co-detected virus. However, no significant differences in clinical characteristics and laboratory findings were found between patients with single HAdV infection and those co-infected with RSV except that co-infections were more frequently observed in younger children. Similarly, a study from Peru also did found no higher prevalence of any clinical manifestations in co-infected patients than in those infected with HAdV alone [26]. The results of another report from Chile showed the clinical severity to be the same in patients with single HAdV infection and those with mixed RSV-HAdV infections [39]. These data demonstrate that, as more sensitive molecular methods become more frequently used to identify pathogens, co-detection of different viruses in the same specimen may also become more common. However, the clinical role of such co-infections will still require independent investigations.

Both HAdV-B7 and HAdV-B3 may cause severe or even fatal pneumonia in even immunocompetent children. Several previous studies showed that patients infected with HAdV-B7 tend to have higher case-fatality rates than those with HAdV-B3 [40, 41]. Two fatal cases were recorded during the study period, and both of these patients were infected with HAdV-B7 alone. Analysis revealed that patients with HAdV-B7 infection had longer duration of fever and higher serum levels of muscle enzymes than HAdV-B3-infected patients. Patients with HAdV-B7 infection also tended to require longer hospital stays although no significant difference was found. These differences have excluded the possible interference by any other co-infected respiratory viruses since this work only evaluated the patients with HAdV infection alone. Such results may suggest that HAdV-B7 infection tended to cause more extrapulmonary tissue damage (such as liver and heart) and may have more severe clinical consequence.

This is a cross-sectional study. Only one respiratory sample was collected from each patient and no viral load analysis was performed. Although HAdV is a pathogen that for long has been known to cause respiratory tract infection, asymptomatic carriage of the virus may persist for weeks [18]. The detection of HAdV in nasopharyngeal aspirate or throat swab with the use of a PCR assay could represent convalescent-phase shedding, so detection may not suggest the current infection.

## Conclusions

In summary, a total of 11 different types of HAdV were identified in children with ALRTIs and HAdV-B7 and HAdV-B3 were the most predominant types. Clinical entities of patients with single HAdV infection were similar to those with mixed HAdV/RSV infections. HAdV-B7 infection tends to have more severe clinical consequences. The presence of newly emerging types or variants and co-infection with different types of HAdV highlights the need for constant and close surveillance of adenovirus infection.

## Abbreviations

HAdV: Human adenoviruses
ALRTIs: Acute lower respiratory tract infections
PIV: Parainfluenza virus
IFV: Influenza virus
RSV: Respiratory syncytial virus
HRV: Human rhinovirus
EV: Enterovirus
HCoV: Human coronavirus
HMPV: Human metapneumovirus
HBoV: Human bocavirus
WBC: White blood cell
CRP: C-reaction protein
AST: Aspartate aminotransferase
ALT: Alanine aminotransferase
LDH: Lactate dehydrogenase
HBDH: Hydroxybutyrate dehydrogenase
